# On the Importance of Keeping a Case-book and Reporting Cases1Read before the annual meeting of the American Veterinary Medical Association, New York, September, 1899.

**Published:** 1899-09

**Authors:** John J. Repp

**Affiliations:** Station Veterinarian, Iowa State College, Ames, Iowa


### ON THE IMPORTANCE OF KEEPING A CASE-BOOK AND REPORTING CASES.[1]

By John J. Repp, V.M.D.,
STATION VETERINARIAN, IOWA STATE COLLEGE, AMES, IOWA.

The part of my subject which I wish to emphasize especially is the latter part; but as it is necessary to keep a case-book in order that an intelligent report of a case can be made, I shall speak of that feature first.

A case-book is a book in which the veterinarian keeps a record of the cases which he treats. For the purpose there may be used a well-bound blank-book, ruled or unruled, and provided with a few pages for indexing names of owners. This book may be provided with a canvas or oil-cloth cover, so that it may be carried on visits without injury. The case-book should be taken along on each visit and the record made at the time of examination. When it is not at hand, a few notes should be made and the subject elaborated on return to the office.

There should be entered in the case-book (*a*) the description of the patient; (*b*) history of patient, including previous illness; (*c*) environment; (*d*) history of present illness, including treatment, if any, given by owner or others; (*e*) etiology; (*f*) present symp-

[1] Read before the annual meeting of the American Veterinary Medical Association, New York, September, 1899.

toms, including what is learned from particular examination of the region complained of, and of all the vital organs and functions of the body; (*g*) methods of diagnosis; (*h*) diagnosis; (*i*) prognosis; (*j*) detailed description of treatment; (*k*) results of treatment; (*l*) history subsequent to illness; (*m*) results of macroscopical, microscopical, and bacteriological examination post-mortem.

This outline should not be printed in the blank-book, but should be carried in the memory, and the record made in accordance with it; the book should be the simplest possible; a printed outline is undesirable. The requirements of each case so vary that headings and subheadings are misleading rather than helpful, and often divert the attention to unimportant details. In addition, any peculiarities not included in the usual outline should be made a matter of record. All these details are essentials in the production of a case-record of value, and none should be omitted.

The record should be faithful; mistakes as well as right judgment and good care should be recorded. A garbled record defeats its own object; it should be technical and scientific. Here the veterinarian should practise the use of scientific rather than vulgar terms. The sentences should be concise, yet their meaning unmistakable. The record should be absolutely private. The privilege of reading it should not be granted to anyone.

The advantages which accrue from a systematic and technical making of case-records are:

1. It engenders more careful and systematic observation, which will lead to a more reliable diagnosis, make the practitioner more accurate, and increase his ability. A careful man is his own severest critic, and if he writes things down, even for his own eye only, he will be more exact than he would otherwise be.

2. It enables the veterinarian to treat his case more skilfully. He always has the carefully recorded facts of the case before him, and is furnished with material of much more value than he would have if he relied solely on his memory. The patient is not subject to the hazard of guesswork on the part of the practitioner during the progress of the disease. A clear record enables the practitioner to study his case at leisure and to work out the meaning of a symptom or group of symptoms by a purely intellectual process.

3. It furnishes material for comparison with conditions under present observation, the teachings of text-books, and the dicta of current literature, so that profit may be derived in treating a patient in hand from the error or the merit of the treatment of a patient that suffered in a similar way at some previous time. This is especially valuable in cases of a new disease.

4. As records accumulate the types of disease in a given locality may be studied and the most efficient therapeutics learned by an experience which, if not formally set down, is in the main forgotten.

5. All successful and eminent practitioners of human medicine habitually keep a case-record. They recognize such practice as essential to the highest grade of work. If we are desirous of obtaining the recognition their profession has secured we must make use of their methods.

6. A case-book, well kept, affords one the consciousness of a duty well and satisfactorily done, of having accomplished something that is above the ordinary. It is a point of differentiation between quackery and the scientific practice of medicine. One of the greatest satisfactions of life is the reflection that something good has been done, and a series of case-books is something that can in later life be pointed to with pride as a monument to industry and painstaking care.

7. If a case-record is kept as it ought to be there will always be at hand, in convenient and reliable form, material to be utilized in reporting cases. And this brings me to the second and perhaps the more important part of my subject.

The cases reported should be those which are somewhat out of the regular routine. There should be something about them which is unique and which teaches something new. Yet we should not be too critical in this respect; for, in order to discover a rule or principle, numbers of observations of a similar nature are required; hence we need not refrain from reporting a condition because some one else has reported it before. Writers judge of the frequency of occurrence of a given condition by the number of cases found recorded in literature. They say a condition is rare if they find it infrequently reported. It may be that in the aggregate the condition is frequent, but observations to this effect have not been put on record. Conditions that are recorded as rare should by all means be reported. Even if a case seems quite trivial and of but little importance to the observer, he should not on this account refrain from putting it on record. A report which may be a matter of but little concern to the one who writes it may be the tenderest and sweetest morsel to the author who is earnestly endeavoring to fill up his outline on some branch of veterinary medicine. It is the little things that give magnificence to science when viewed as a finished product.

Briefly, the benefits which may be obtained by reporting cases to our journals of veterinary medicine and to veterinary medical societies for publication in the report of their proceedings are:

1. If an intelligent report of a case is made it serves to introduce the writer in a favorable manner to those who read the article, and to bring him prominently before the profession. He will in this way acquire that standing among professional men that all good veterinarians deserve.

2. It renders the veterinarian more alert to detect new forms of disease.

3. Other practitioners will find careful reports of incalculable value to them in their treatment of cases which they meet in their practice. It gives them a "lamp of experience" to guide them. They are thus relieved of the necessity of resorting to makeshift methods, the efficiency of which they can only conjecture. It is well to have the power of original device in case of need, but no one should be compelled to depend upon his own untried methods because he is deprived of the experience of his predecessors and contemporaries.

4. It affords writers of articles on technical medical subjects data which they may use for the purpose of substantiating some principle they have evolved, or give them facts from which they may make original inductions.

5. It is only through the agency of reports of cases that we will be able to build up a national veterinary medical literature. These reports will furnish to those who write our books the larger part of the material which is necessary to book-making. Writers, in order to produce a valuable scientific work, must have a large collection of reliable records which have been contributed by the rank and file of the profession for a long period of years. A celebrated surgeon of Philadelphia recently wrote on this point: "The report of cases bears the same relation to a national medical literature as does the farmer to the body politic, viz., it is the foundation—the backbone. There is scarcely a single important contribution to medical science which is not based on the report of cases. The student collects these cases from general literature and draws from this collection deductions which are useful to the whole profession. Case-reports may be considered the springs, which, if sufficiently numerous, make the mighty river of progress of medical science."

The entire fabric of science is but the recorded experience of individuals. In the words of another: "Science is 'knowledge gained by systematic observation, experiment, and reasoning coördinated, arranged, and systematized.' "

By this I do not mean that anyone who has this material can produce a technical and worthy treatise. The writer of a book should be a man of high attainment and wide experience, so that he may possess wise judgment, close discrimination, and high powers of reasoning. He must be on a plane far above the mere compiler; yet no one can write a treatise on veterinary medicine worth reading without making frequent and constant use of the experience and reasonings of others.

It is in a large measure the character of the writer that makes the difference between the compiled and the elaborated treatise. The compiler and the elaborator both use the experience of others; but the former jumbles that experience together in a careless manner and makes use of but little original thought or experience, while the latter uses the experience of others with discretion, and disposes it according to his own wise judgment and broad education. It is for the use of men of the latter class that the current magazines and periodicals and reports of societies should be teeming with reports of experiences and investigations of the practitioner and experimenter.

It is a fact much to be deplored that there is so little of scientific veterinary literature by American authors. Practitioners here are compelled to rely in a large measure upon foreign publications or their translations. The Germans and French have excellent treatises on the various branches of veterinary medicine, well suited for their own veterinarians, but these works are in many instances not adapted to the needs of the American practitioner. America has such immense live-stock interests that she should be represented by a bountiful literature covering every phase of the subject and kept revised by the addition of the latest developments of science. The demand for such a literature is imperative. Our sister profession has accomplished this task, and if we wish to obtain such recognition as we ought to have from the fraternity of human medicine we must produce an adequate literature.

European writers have had more than a hundred years of active scientific veterinary experience and research from which to draw in accomplishing their excellent literary labors. In America we have had scarcely more than four decades, during which entirely too little writing has been done by our practitioners. I do not believe that it is possible at the present time for us here in America to produce an adequate set of treatises on veterinary subjects from material which is in sufficiently large measure our own, for such material is not yet in existence. What we need is a score of years of activity with the pen on the part of our practitioners, recording, whenever occasion requires, their individual research and experience. At the end of that time I believe we can produce a literature which will be equal to that of the European nations and compare favorably with that of human medicine.

An eminent physician recently wrote: "There can be no broad, useful medical literature without careful reports and analyses of large numbers of cases. Such literature can only be built up by reporting accurate observations upon all phases of disease under all the conditions in which it is met."

That excellent quotation used in the *American Veterinary Review*, and which Dr. Bell writes me is to be credited to the *Veterinary Record*, says: "Careful observation makes a skilful practitioner, but his skill dies with him. By recording his observations he adds to the knowledge of his profession, and assists by his facts in building up the solid edifice of pathological science."

Let us unite in a persevering effort to build up a national veterinary medical literature.

